# Altered Functional Network Connectivity of Precuneus and Executive Control Networks in Type 2 Diabetes Mellitus Without Cognitive Impairment

**DOI:** 10.3389/fnins.2022.887713

**Published:** 2022-06-27

**Authors:** Jinjian Wu, Shangyu Kang, Jianpo Su, Kai Liu, Liangwei Fan, Xiaomeng Ma, Xin Tan, Haoming Huang, Yue Feng, Yuna Chen, Wenjiao Lyu, Lingli Zeng, Shijun Qiu, Dewen Hu

**Affiliations:** ^1^The First School of Clinical Medicine, Guangzhou University of Chinese Medicine, Guangzhou, China; ^2^College of Intelligence Science and Technology, National University of Defense Technology, Changsha, China; ^3^Department of Radiology, The First Affiliated Hospital of Guangzhou University of Chinese Medicine, Guangzhou, China

**Keywords:** type 2 diabetes mellitus, cognitive impairment, precuneus, functional magnetic resonance imaging, network connectivity

## Abstract

In epidemiological studies, type 2 diabetes mellitus (T2DM) has been associated with cognitive impairment and dementia, but studies about functional network connectivity in T2DM without cognitive impairment are limited. This study aimed to explore network connectivity alterations that may help enhance our understanding of damage-associated processes in T2DM. MRI data were analyzed from 82 patients with T2DM and 66 normal controls. Clinical, biochemical, and neuropsychological assessments were conducted in parallel with resting-state functional magnetic resonance imaging, and the cognitive status of the patients was assessed using the Montreal Cognitive Assessment-B (MoCA-B) score. Independent component analysis revealed a positive correlation between the salience network and the visual network and a negative connection between the left executive control network and the default mode network in patients with T2DM. The differences in dynamic brain network connectivity were observed in the precuneus, visual, and executive control networks. Internal network connectivity was primarily affected in the thalamus, inferior parietal lobe, and left precuneus. Hemoglobin A1c level, body mass index, MoCA-B score, and grooved pegboard (R) assessments indicated significant differences between the two groups (*p* < 0.05). Our findings show that key changes in functional connectivity in diabetes occur in the precuneus and executive control networks that evolve before patients develop cognitive deficits. In addition, the current study provides useful information about the role of the thalamus, inferior parietal lobe, and precuneus, which might be potential biomarkers for predicting the clinical progression, assessing the cognitive function, and further understanding the neuropathology of T2DM.

## Introduction

Aging, overeating, a sedentary lifestyle, and lack of physical exercise have led to the present high prevalence of prediabetes and diabetes. Currently, 463 million people worldwide live with type1 or type 2 diabetes mellitus (T2DM), especially in low- and middle-income countries, and 1.8 million people die from this disease annually. Moreover, more than 50 million people live with dementia, and this number is expected to increase threefold by the year 2050 (Gregg et al., 2016; Advocacy guide to the IDF Diabetes Atlas, 2019; World Alzheimer Report, 2019; Beeri and Bendlin, 2020; Biessels et al., 2020). As both the number of cases and the prevalence of diabetes have been steadily increasing over the past few decades (Biessels and Despa, 2018), cognitive impairment and dementia have developed into a growing global public health concern owing to population aging (Lehtisalo et al., 2016; Callisaya et al., 2019; Sneag and Tan, 2020). Previous research examining the association between prediabetes and cognitive decline is limited and often reports a lack of an association. However, diabetes has been reported as a risk factor for dementia in some studies (Spauwen et al., 2013; Marseglia et al., 2019; Yu et al., 2019; Ganguli et al., 2020; Li et al., 2020, 2021).

T2DM, which accounts for approximately 60% of all diabetic cases, primarily characterized by hyperglycemia, insulin resistance, and relative insulin deficiency. Accumulation of advanced glycation end products (AGEs) may lead to inflammation, oxidative stress, protein cross-linking, cerebral insulin resistance and metabolic disorders (Brownlee, 2001; Wei et al., 2015; Yang et al., 2016), damage small and large blood vessels, thereby leading to disabling complications including retinopathy, nephropathy, neuropathy, and cardiovascular and cerebrovascular diseases (Zeng et al., 2018; Liu et al., 2019; Li et al., 2020). Multiple population studies have consistently shown an increased risk of dementia in patients with T2DM (Kashyap et al., 2019). Before dementia onset, T2DM is associated with deficits in different cognitive domains (Biessels et al., 2014), particularly with impairments of psychomotor speed and executive function (Marseglia et al., 2018). However, other prospective studies have failed to confirm such associations (Reijmer et al., 2013; Kashyap et al., 2019). Because very few studies have investigated the association between prediabetes and cognitive impairment, the available evidence is not conclusive (Callisaya et al., 2019; Herbet et al., 2019; Yu et al., 2019).

Previous cross-sectional magnetic resonance imaging (MRI) studies have consistently reported associations between T2DM and reduced global brain volume (Wardlaw et al., 2013; Weinstein et al., 2015; Zhang et al., 2020; Adachi et al., 2021; Hyung et al., 2021). In addition, microvascular lesions and neurodegeneration markers related to Alzheimer’s disease (AD) have been associated with T2DM (Tan et al., 2019; Zhang et al., 2019; Wu et al., 2020). A few longitudinal studies on the progression of cerebral microvascular lesions or AD-related neurodegeneration in T2DM yielded inconsistent findings (Zhang et al., 2018). Cerebral small vessel, disease including white matter hyperintensity and brain atrophy, is a major cause of vascular dementia (Reijmer et al., 2013), whereas atrophy of the medial temporal lobe (containing the hippocampus) is a marker of neurodegeneration in AD (Huntley et al., 2017). Although the association between diabetes and dementia is well established, it is not known whether type 2 diabetes with no or early stage cognitive impairment is associated with vascular or AD-related neurodegenerative mechanisms.

To date, although there are numerous previous T2DM studies on differences in functional connectivity (FC) (Wang et al., 2019), especially in default mode network (Yang et al., 2016; Croosu et al., 2022) which is the main impaired network in T2DM patients, internal and external network connectivities in other key cognitive-related networks have not been fully investigated. We evaluated static-dynamic network connectivity and internal network connectivity in order to fully characterize the differences between patients with T2DM without cognitive impairment (T2DM-NCI) and normal controls (NCs). Since it is difficult to distinguish diabetic patients with cognitive impairment from those with AD, patients with cognitive impairment are commonly excluded in T2DM diabetes studies, and only patients without cognitive impairment and NCs are investigated. We aimed to identify the key differences between patients with T2DM-NCI and NCs to obtain an in-depth understanding about the progression of memory and cognitive impairments in T2DM.

## Materials and Methods

There were 90 patients with T2DM and 76 healthy individuals agreed to participate in this study (September 2018–March 2021), we had excluded subjects with brain tumors (*n* = 2), missing MoCA-B scores (*n* = 12), developmental disorders (*n* = 2), or neuropsychiatric diseases (*n* = 2) (e.g., Parkinson’s disease or schizophrenia), a total of 82 patients with T2DM and 66 healthy individuals were eventually enrolled in the study. This study was approved by the ethics committee of The first affiliated hospital of Guangzhou University of Chinese Medicine and written informed consent was obtained from all subjects. Subjects with T2DM were selected from among hospitalized patients and outpatients at the Endocrinology Department of The First Affiliated Hospital of Guangzhou University of Chinese Medicine, while NCs were recruited as volunteers over the same period. T2DM was diagnosed when fasting blood glucose was >7.0 mmol/L on two separate occasions or when the 2-h blood glucose level was >11.1 mmol/L during a 75-g oral glucose tolerance test (Wass and Stewart, 2011).

The exclusion criteria for both groups were as follows: cognitive impairments, impaired glucose tolerance or impaired fasting glucose (Ogurtsova et al., 2017) that not diagnosed as T2DM yet, serious eye diseases, any sign of positive neurological symptoms, any history of neurological abnormality, serious head injuries (with loss of consciousness >5 min), left- or mixed-handedness, body mass index (BMI) >31 kg/m^2^, substance (alcohol, tobacco, psychoactive drug) abuse, moderate-severe hypertension [systolic blood pressure (SBP) = 150 mmHg or diastolic blood pressure = 100 mmHg] or mild hypertension took medication for 3 months prior to the start of the study and during the study, severe hypoglycemia or hyperlipemia, specific abnormalities found on conventional MRI scans, or other factors that might affect brain structure and function (chronic infections, organic failure, and other endocrine diseases).

### Clinical, Biochemical, and Neuropsychological Assessments

Body weight and height were measured without shoes or heavy clothes. BMI was calculated as weight in kilograms divided by the squared height in meters (kg/m^2^). Arterial blood pressure was measured on the both arms in a sitting position after rested for 5 min, and hypertension blood pressure was identified (blood pressure 140/90 mmHg).

Cognition, global cognition, cognitive ability, short-term and long-term word memory, attention and short-term number memory, attention and neuromotor speed, number-to-character conversion and speed writing skills, and finger dexterity for both handedness and non-handedness were assessed using the Mini-Mental State Examination (MMSE), MoCA-B (patients with a MoCA-B score <26 were diagnosed with mild cognitive impairment) (Schrag et al., 2017; Li et al., 2018), MMSE, World Health Organization University of California-Los Angeles auditory verbal learning test (AVLT), digit span test (DST), trail-making test (TMT), digit symbol substitution test (DSST), and grooved pegboard, respectively.

### Magnetic Resonance Imaging Data Acquisition

All images were acquired on a Siemens (Munich, Germany) 3T Prisma scanner using a standard 64-channel head coil. Functional volumes were acquired using a multiple slice T2-weighted echo planar imaging sequence with the following parameters: repetition time, 2,000 ms; echo time, 30 ms; flip angle, 90°; matrix dimensions, 64 × 64; field of view, 100 mm; slice thickness, 3.5 mm; and number of slices, 33. High-resolution three-dimensional T1-weighted images were acquired using the magnetization-prepared rapid gradient-echo sequence with the following parameters: repetition time, 2,530 ms; echo time, 2.98 ms; inversion time, 1,100 ms; flip angle, 7°; field of view, 256 × 224 mm^2^; matrix size, 224 × 256 interpolated to 448 × 512, 192 sagittal slices; slice thickness, 1.0 mm; and voxel size, 0.5 × 0.5 × 1 mm^3^.

### Preprocessing

Preprocessing and statistical analyses of imaging data were performed with SPM12 (Wellcome Department of Imaging Neurosciences, University College London, United Kingdom)^1^ and GRETNA (GRETNA v2.0)^2^ under Matlab R2013b. All functional images were first corrected for slice timing with the middle slice in the acquisition order as the reference and realigned to the first image to correct head movement. Thirteen subjects showed >2-mm translation and >2° rotation in the localizer task; thus, their data were eliminated from the localizer analysis. Individual functional images were co-registered to the mean functional image. The T1 image was coregistered to a mean functional image and then segmented. The functional images were then normalized to the Montreal Neurological Institute (MNI) space using DARTEL after the segmentation. An anisotropic 6-mm full-width half-maximum Gaussian kernel was used for spatial smoothing.

The preprocessing procedures included slice timing correction, within-subject interscan realignment to correct for possible movement, spatial normalization to a standard brain template in the MNI coordinate space, resampling to a 3 × 3 × 3-mm^3^ voxel size (Berman et al., 2016), and smoothing with a 6-mm full-width half-maximum Gaussian kernel. Finally, the functional data were high-pass filtered with a cutoff frequency of 0.01–0.08 Hz (Yeo et al., 2011).

### Statistical Analyses

Individual activation maps were generated in the first-level analysis using the general linear model in SPM8 (see text footnote 1). Condition onset and duration were convolved with the canonical hemodynamic response function (HRF). Realignment parameters were included in the design matrix as covariances to regress out the movement-related variance.

To perform the functional network connectivity analysis, we first conducted a spatial group independent component analysis (ICA) to extract the functional network of the brain using the Infomax algorithm in GIFT (Group ICA of functional MRI Toolbox v3.01).^3^ The number of independent components was estimated using the minimum description length approach, resulting in an average of 41 independent components. The preprocessed resting-state data of all subjects were temporally dimension-reduced using PCA and concatenated. All subjects were then concatenated into one group and passed through another dimension reduction step. The grouped data were decomposed into 20 aggregate components (Hutchison et al., 2013). Then a GICA back reconstruction was performed to calculate the individual subject components (including subject-specific spatial maps and time-courses) based on the aggregate ICA components. Twenty components were identified as meaningful based on the criteria by Allen et al. (2014). Using spatial correlation values between independent components and the template, we then sorted the selected 14 independent components into eight functional networks: left executive control network (LECN), right executive control network (RECN), primary visual network (PVN), extrastriate visual network (EVN), salience network (SN), language network (LN), precuneus network, and default mode network (DMN) (Yeo et al., 2011).

## Results

### Clinical and Neuropsychological Results

The clinical and neuropsychological results of the patients and NCs are summarized in Table 1. Patients and NCs were matched for age, sex, and educational level (*p* > 0.05), and there were significant differences in hemoglobin A1c levels, BMI, MoCA-B scores, and grooved pegboard (R) (*p* < 0.05) between the two groups. There were no significant differences in the other neuropsychological tests results between the two groups (*p* > 0.05) (Table 1).

**TABLE 1:** Clinical and neuropsychological results of patients with type 2 diabetes mellitus (T2DM) and normal controls (NCs).

	*p*	T2DM		NCs	
			
		Mean	*SD*	Mean	*SD*
**Demographic data**					
Age (years)	0.736	46.09	8.17	46.64	11.03
Sex (M/F)	0.900^#^	53/29		42/24	
Education (years)	0.521	11.67	3.68	12.06	3.65
BMI (kg/m^2^)	0.003*	24.52	3.14	23.02	2.76
**Clinical data**					
HbAlc (%)	0.000*	9.295	2.57	4.77	0.62
DBP (mmHg)	0.203	128.73	18.12	124.41	19.23
SBP (mmHg)	0.071	85.93	10.72	82.32	11.18
TG (mmol/L)	0.901	2.38	1.93	NA	NA
TC (mmol/L)	0.535	4.77	0.91	NA	NA
LDL (mmol/L)	0.761	3.12	0.91	NA	NA
FBG (mmol/L)	0.000	8.95	2.98	4.72	0.63
FINS (uIU/mL)	NA	11.84	11.60	NA	NA
mALB (mg/L)	NA	28.87	81.08	NA	NA
ACR (mg/g)	NA	28.58	95.37	NA	NA
24 h UPRO (g/24 h)	NA	0.20	0.23	NA	NA
M-TP (mg/L)	NA	102.57	64.50	NA	NA
C-Peptide (ng/mL)	NA	2.09	1.10	NA	NA
**Neuropsychological data**					
AVLT (immediate)	0.823	23.47	4.90	23.67	5.26
AVLT (5 min)	0.999	8.42	2.36	8.54	2.47
AVLT (20 min)	0.357	9.27	2.41	9.27	2.27
AVLT (recall)	0.348	10.74	2.76	11.13	1.87
DST (direct)	0.646	7.71	1.49	7.83	1.34
DST (reverse)	0.795	4.99	1.46	4.92	1.55
TMT-A	0.447	50.23	20.51	52.89	20.23
TMT-B	0.914	42.71	18.95	43.05	16.79
Clock	0.893	3.51	0.73	3.49	0.62
MoCA-B	0.006*	26.29	2.92	27.33	1.47
MMSE	0.877	28.19	1.99	28.24	1.72
DSST	0.848	48.84	13.52	48.68	15.36
Grooved pegboard (R)	0.017*	75.17	15.85	68.63	15.96
Grooved pegboard (L)	0.256	82.69	20.82	78.66	20.79

*Data are shown as mean ± standard deviation, independent sample t-tests.*

^#^Pearson’s Chi-square test (2-sided), *p < 0.05 indicates statistically significant differences. BMI, body mass index; DBP, diastolic blood pressure; SBP, systolic blood pressure; TG, triglyceride; TC, total cholesterol; LDL, low-density lipoprotein; FBG, fasting blood glucose; FINS, fasting serum insulin; mALB, microalbuminuria; ACR, albumin/creatinine ratio; 24 h UPRO, 24-h urinary protein; M-TP, micro total protein; AVLT, auditory verbal learning test; DST, digit span test; TMT-A, trail making test-A; TMT-B, trail making test-B; MoCA-B, Montreal Cognitive Assessment-B; MMSE, Mini-Mental State Examination; DSST, digit symbol substitution test.

### Independent Component Analysis

**FIGURE 1:**
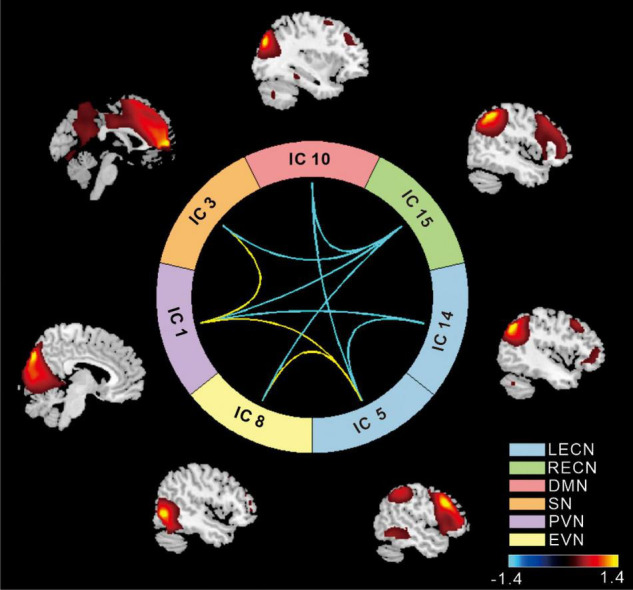
The cool color scale represents negative values, indicating hypoconnectivity (decreased positive correlation, or greater anti-correlation) in patients with type 2 diabetes mellitus (T2DM) relative to normal controls (NCs); the hot color scale represents positive values, indicating hyperconnectivity (increased positive correlation or less anti-correlation) in patients with T2DM relative to NCs. LECN, left executive control network; RECN, right executive control network; PVN, primary visual network; EVN, extrastriate visual network; SN, salience network; DMN, default mode network (*p* < 0.05, false discovery rate-corrected).

Based on the ICA, the following networks were selected for further analysis: the left executive control network (LECN), the right executive control network (RECN), the primary visual network (PVN), the extrastriate visual network (EVN), the salience network (SN), and the default mode network (DMN). Compared to the NC group, in the T2DM group high positive connectivity was mainly observed between the SN with the PVN and the LECN with the EV (*p* < 0.05), whereas negative connectivity was mainly observed between the RECN with the SN, DMN, PVN, EVN, and LECN with the DMN (*p* < 0.05) (Figure 1).

### Functional Internal and External Network Connectivity Analysis

**FIGURE 2:**
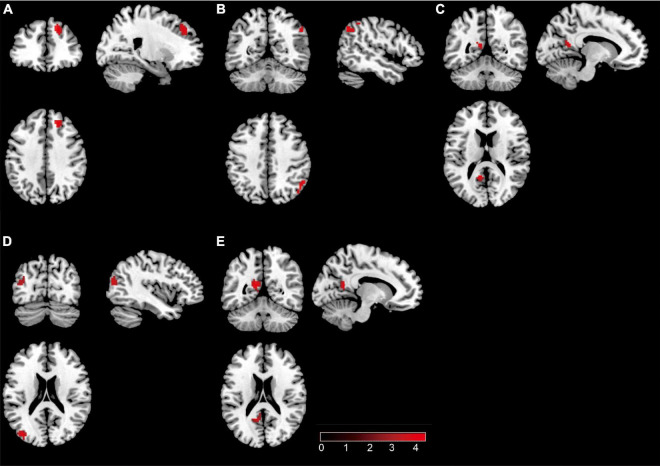
The brain regions with a significant difference in patients with type 2 diabetes mellitus. **(A)** Significant difference in internal network connectivity in the SN. **(B)** Significant difference in internal network connectivity in the LECN. **(C)** Significant difference in internal network connectivity in the DMN. **(D)** Significant difference in internal network connectivity in the SN. **(E)** Significant difference in internal network connectivity in the precuneus network. The significance threshold was set at *p* < 0.05 (false discovery rate-corrected).

Internal network connectivity was significantly different in the SN, LECN, DMN, VN, and precuneus network [*p* < 0.05, false discovery rate (FDR)-corrected] (Figure 2). The main internal network connectivity differences in the SN were observed in the right upper frontal lobe, those in the LECN were observed in the right angular and right inferior parietal lobe, those in the DMN were observed in the left precuneus, those in the VN were observed in the left superior parietal and occipital lobes, and those in the precuneus network were observed in the left precuneus and left posterior cingulate. The analysis showed significant differences in all these regions (Table 2).

**TABLE 2:** Abnormal internal network connectivity in patients with type 2 diabetes mellitus compared to normal controls.

Network	Brain region	Peak MNI coordinates			Voxel (mm^3^)	t	z
		
		x	*y*	z			
SN	Frontal_Sup_2_R	18	39	42	52	4.49	4.33
LECN	Parietal_Inf_R	51	–57	39	28	4.10	3.99
	Angular_R	45	–69	39	27	4.41	4.27
DMN	Precuneus_L	–12	54	18	20	4.44	4.29
VN	Parietal_Sup_L	–24	–69	54	10	3.87	3.77
	Occipital_Sup_L	–36	–81	24	19	5.37	5.12
Precuneus network	Precuneus_L	–12	–54	39	25	5.02	4.81
	Cingulate_Post_L	–12	–55	24	10	3.55	3.57

*Group differences in network internal connectivity were evaluated using two-sample t-tests, post hoc analysis based on two-sample two-sided t-tests were used to assess the significant effects (p < 0.05, false discovery rate-corrected). MNI, Montreal Neurological Institute; L, left; R, right.*

**FIGURE 3:**
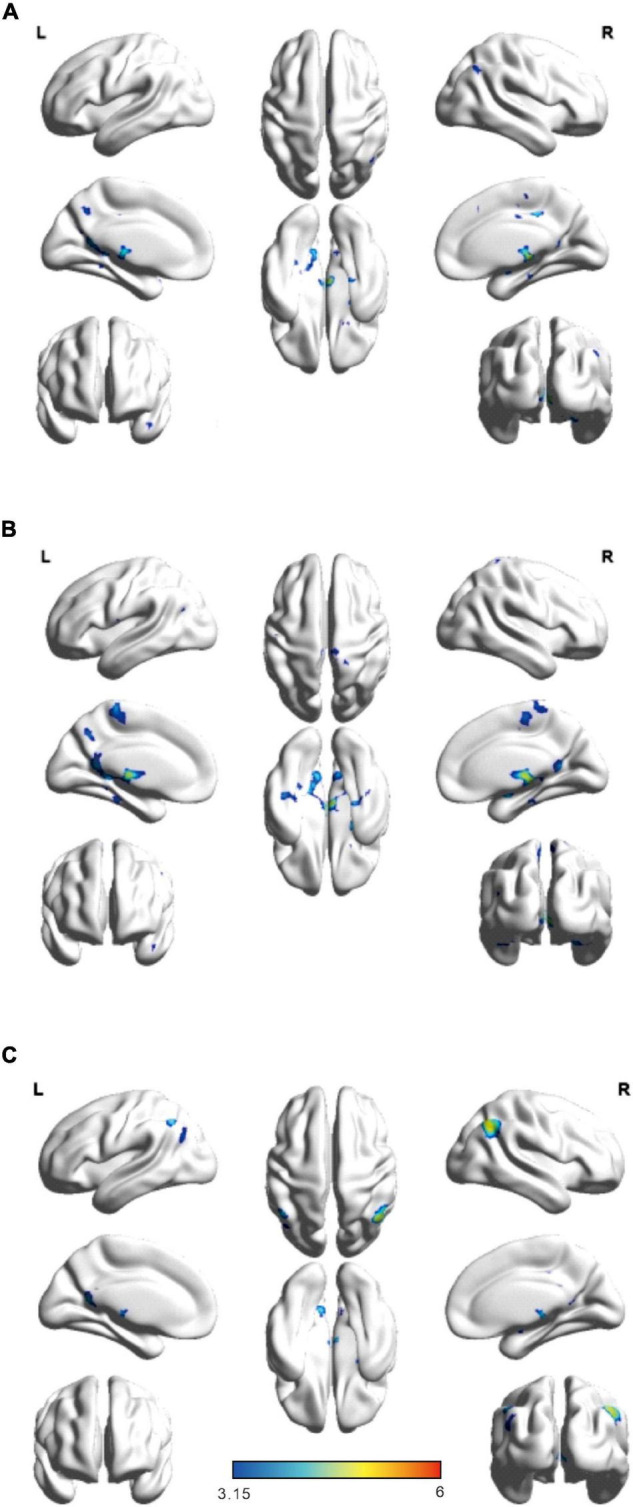
Significant brain network regions are rendered on the surface of the automated anatomical labeling atlas in BrainNet Viewer. **(A)** Significant difference network connectivity in the left executive control network with the whole brain. **(B)** Significant difference network connectivity in the right executive control network with the whole brain. **(C)** Significant difference network connectivity in the precuneus network with the whole brain. The significance threshold was set at *p* < 0.05 (false discovery rate-corrected, cluster size > 20). R, right; L, left.

External network connectivity was observed between the network and whole brain, and its analysis showed that the LECN, RECN, and precuneus network had significant differences (*p* < 0.05, FDR corrected). Subsequently, to reveal the regions specifically related to these three networks, they were selected as targets for whole-brain FC. Compared to the NC group, the T2DM group showed differences in the right middle cingulate, left paracentral lobule, right angular, left inferior parietal, right middle temporal, and right inferior temporal lobes, left superior anterior cingulate cortex, right precuneus in the LECN; right superior temporal, left middle temporal lobes, and left thalamus in the RECN; right angular, left middle occipital, left inferior parietal lobe, and right precuneus in the precuneus network (*p* < 0.05, FDR-corrected, Figure 3 and Table 3).

**TABLE 3:** Abnormal external network connectivity in patients with type 2 diabetes mellitus compared to normal controls.

Network	Brain region	Peak MNI coordinates			Voxel (mm^3^)	t	z
		
		x	*y*	z			
LECN							
	Cingulate_Mid_R	1	–30	50	133	4.81	4.62
	Paracentral_Lobule_L	3	–33	78	81	4.81	4.63
	Angular_R	51	–63	39	79	4.41	4.26
	Parietal_Inf_L	–48	60	48	59	3.70	3.61
	Temporal_Mid_R	66	–48	6	42	4.32	4.18
	Temporal_Inf_R	54	–60	–12	34	4.04	3.92
	ACC_sup_L	3	42	15	30	4.19	4.07
	Precuneus_R	12	–54	12	25	4.07	4.96
	Occipital_Mid_L	–51	–75	9	24	3.55	3.47
	Temporal_Pole_Mid_L	–42	15	–24	24	3.73	3.64
	Cerebellum_9_L	–6	–57	–42	22	3.91	3.81
RECN							
	Paracentral_Lobule _L	3	–30	78	185	5.19	4.96
	Temporal_Sup_R	63	0	–9	104	4.56	4.40
	Temporal_Mid_L	–48	–69	21	57	4.06	3.97
	Thal_PuM_L	–7	–26	4	50	6.58	6.14
	Cerebellum_9_R	18	–45	–45	46	3.95	3.84
	Temporal_Mid_R	66	–51	3	45	4.10	3.98
	Angular_R	48	–69	48	43	4.01	3.90
	Occipital_Mid_L	44	–69	15	41	3.11	3.05
	Temporal_Pole_Mid_L	–39	21	–30	32	3.84	3.74
Precuneus network							
	Angular_R	50	–57	42	243	5.35	5.11
	Parietal_Inf_R	54	–60	39	133	5.63	5.34
	Angular_L	–45	–64	41	114	4.69	4.52
	Parietal_Inf_L	–48	–60	45	90	4.69	4.52
	Precuneus_R	–21	27	9	66	5.25	5.02
	Caudate_R	15	–6	21	51	4.54	4.38
	Cerebellum_Crus2_L	–39	–75	–51	44	3.93	3.82
	Frontal_Mid_2_L	–48	21	45	26	3.77	3.68

*Group differences in functional network internal connectivity were evaluated using two-sample t-tests, post hoc analysis based on two-sample two-sided t-tests were used to assess the significant effects (p < 0.05, false discovery rate-corrected, cluster size > 20). MNI, Montreal Neurological Institute; L, left; R, right.*

### Group Differences in Dynamic Functional Network Connectivity

**FIGURE 4:**
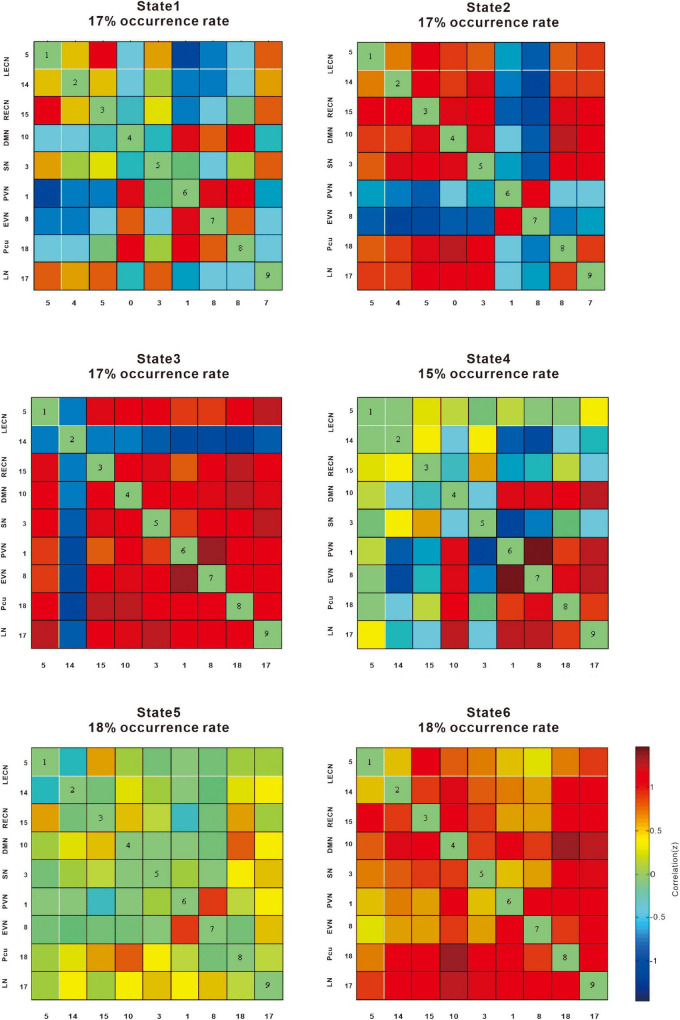
The correlation (z) provides information on the effect size. The cool color scale represents negative values, indicating hypoconnectivity (decreased positive correlation, or greater anti-correlation) in patients with type 2 diabetes mellitus (T2DM) relative to normal controls (NCs); the hot color scale represents positive values, indicating hyperconnectivity (increased positive correlation or less anti-correlation) in patients with T2DM relative to NCs. LECN, left executive control network; RECN, right executive control network; Pcu, precuneus network; PVN, primary visual network; EVN, extrastriate visual network; LN, language network; SN, salience network, DMN, default mode network.

The six dynamic states are depicted in Figure 4. We focused on connectivity differences between the groups (T2DM vs. NC). We identified the connectivity between the LECN, RECN, precuneus network, PVN, EVN, LN, SN, and DMN on the basis of the reduced models of states described in previous studies the connectivity between networks LECN, RECN, precuneus network, PVN, EVN, language network (LN), SN, and DMN was identified (Figure 4).

State 1 showed a negative correlation between the precuneus network, PVN, and EVN, whereas the DMN, LECN, and RECN were positively correlated in patients with T2DM compared with NCs.

State 2 showed a negative correlation between the EVN and PVN, whereas the SN, DMN, and LECN were positively correlated in patients with T2DM compared with NCs.

State 3 showed a negative correlation with the LECN, whereas the LN, precuneus network, EVN, PVN, SN, DMN, and RECN showed a positive correlation in patients with T2DM compared with NCs.

State 4 showed a negative correlation between the LECN and EVN, PVN, whereas the LN, precuneus network, EVN, and PVN showed a positive correlation in patients with T2DM compared with NCs.

State 5 showed an increased positive correlation between the EVN and PVN, while the other networks were not significantly weakened.

State 6 showed that overall connectivity increased positive correlation.

The k-means algorithm was repeated 1,000 times for each k value, and the clustering results were recorded each time. The clustering evaluation results for different cluster numbers are shown in Figure 4. For each *k*-value of 1,000 repeated tests, when the k-mean was 6 in the dynamic functional network connectivity calculation, states 3 and 4 differed significantly (*p* < 0.05, FDR-corrected). In state 3, LN, precuneus network, EVN, PVN, SN, DMN, and RECN connectivity was enhanced, whereas LECN connectivity was weakened. In state 4, LN, precuneus network, EVN, and PVN connectivity was enhanced, whereas connectivity in the LECN with EVN and PVN was weakened. Overall, connectivity in the LN, precuneus network, EVN, and PVN increased, whereas LECN, EVN, and PVN connectivity was lower in the T2DM group compared with the NC group.

### Group Differences in Mean Dwell Time

**FIGURE 5:**
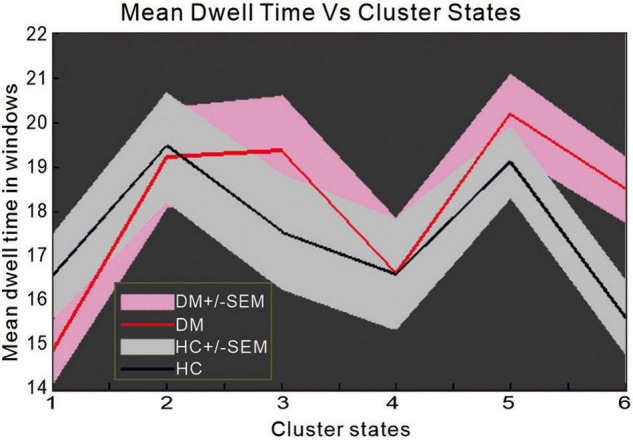
The difference in mean dwell time between patients with type 2 diabetes mellitus and normal controls.

In state 6, we observed significant differences between the two groups in mean dwell time (*p* = 0.0098, *T* = 2.6187) (Figure 5), indicating that patients with T2DM remain longer in state 6 than NCs.

### Clinical Correlate Analysis

**FIGURE 6:**
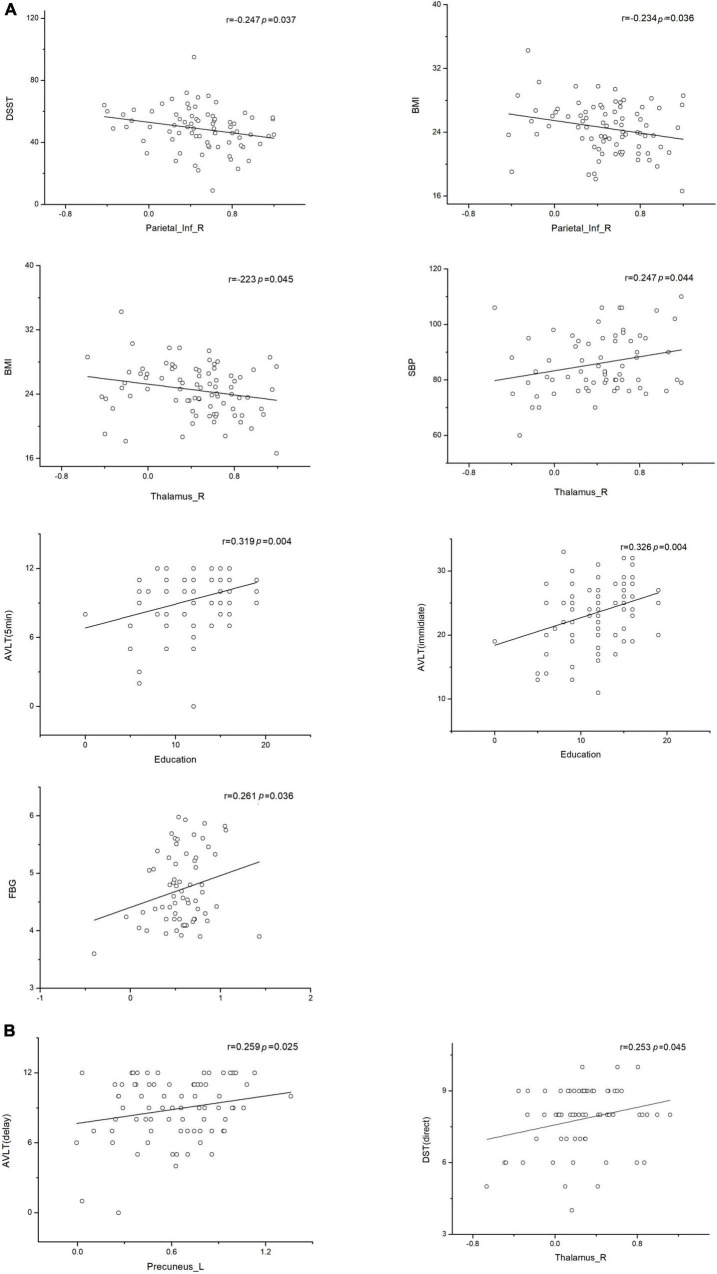
Associations between functional connectivity and neuropsychological test scores. Pearson correlation was conducted on functional connectivity and neuropsychological test scores. **(A)** Correlation of functional connectivity with neuropsychological test scores in the type 2 diabetes mellitus group. **(B)** Correlation of functional connectivity with neuropsychological test scores in the normal control group (*p* < 0.05 indicates statistically significant differences). L, left; R, right.

The correlations between brain network parameters, including significantly different brain regions and cognitive function scores, were analyzed by using Pearson’s correlation for the T2DM and NC groups. In the T2DM group, the left precuneus and right thalamus showed positive correlations with the AVLT (delay) (*r* = 0.259, *p* = 0.025) and SBP (*r* = 0.247, *p* = 0.044), respectively. The right thalamus negatively correlated with BMI (*r* = –0.223, *p* = 0.045). The right inferior parietal lobe showed negative correlations with BMI (*r* = –0.234, *p* = 0.036) and DSST (*r* = –0.247, *p* = 0.037). As for the correlation between scales, educational level showed a positive correlation with the AVLT (immediate) (*r* = 0.326, *p* = 0.004) and the AVLT (5 min) (*r* = 0.319, *p* = 0.004). In the NC group, the left precuneus showed a positive correlation with fasting blood glucose (*r* = 0.261, *p* = 0.036) (Figure 6), and a significant negative association was observed between the FC and the time consumption component of the memory response speed test.

## Discussion

This study found that patients with T2DM-NCI start to exhibit altered brain connectivity in the executive control and the precuneus network, with the main differences, compared with NCs, found in the thalamus, inferior parietal lobe, and precuneus. These observations provide a perspective for follow-up studies in patients with T2DM-NCI. In the present study, we focused on functional network connectivity in patients with T2DM-NCI and NCs. ICA revealed increased connectivity between the SN and VN and decreased connectivity between the LECN and DMN in patients compared to the NC group. These results were further validated by a dynamic network connectivity analysis (Figure 4), which identified differences in connectivity between the two groups mainly in the precuneus network, VN, and LECN. Furthermore, we found higher negativity in the LECN in the T2DM group, while the precuneus network was more active, which may be a compensatory mechanism in T2DM-NCI. Previous studies have often focused on cognitive impairment in patients with T2DM, and found that the main impaired networks are the DMN, SN, and ECN, whereas the DMN appears to be already disrupted before cognitive impairment (Cui et al., 2016; Furler et al., 2017; Zhang et al., 2019; Ali et al., 2020; Croosu et al., 2022). What is more, the DMN has been shown to be mainly involved in impaired intranetwork and internetwork connectivity. Bilateral posterior cerebellum showed duration-associated reduced connectivity strength, which is one of the representative regions of the DMN (Yang et al., 2016). We found significant differences between ECN and precuneus network connectivity, which suggests that the ECN is also damaged before cognitive impairment in patients with T2DM. These findings provide useful evidence for the precuneus network as a sensitive indicator in the DMN of patients with T2DM before cognitive impairment.

We found that the main regions undergoing FC alterations were the inferior parietal lobe, right angular gyrus, left thalamus, left precuneus, left superior parietal and left superior occipital lobes, and left posterior cingulate. The key pathological changes in T2DM-NCI involve the hippocampus, temporal lobe, left inferior parietal lobe, cingulate gyrus, and precuneus (Herbet et al., 2019). Compared to NCs, we found that the main pathological changes affect the same regions that involve thalamus, left precuneus, inferior parietal lobe, and angular gyrus in T2DM-NCI. This suggests that changes in these brain regions may be associated with the onset of cognitive impairment and evolve before cognitive decline in patients with T2DM. In subsequent studies, we will aim to determine the diabetes diagnosis time in patients with T2DM. The disease development may depend on blood sugar levels, thereby affecting the time-course of diabetic encephalopathy. In elderly patients with diabetes, studies have found that chronically reduced insulin secretion, low insulin concentrations, or high glucagon levels can lead to impaired insulin signaling in the brain. In turn, this leads to overproduction of antibodies, hyperphosphorylation of tau, and hippocampal atrophy. These neurodegenerative alterations (Huntley et al., 2017) result in memory loss and cognitive decline and impair self-care abilities. Previous studies have demonstrated that functional connections in the hippocampus, para-hippocampus, frontal lobe, and temporal cortex are abnormal in patients with T2DM (Liang et al., 2019; Tan et al., 2019), which also supports the current findings. However, the main difference between these earlier and our study is that in addition to the commonly observed alterations in the hippocampus and parahippocampal gyrus, we identified other brain regions including the temporal lobe, thalamus, left precuneus, and angular gyrus that are involved in the early disease stage and are more sensitive to the onset of cognitive impairment in patients with T2DM. In addition, the precuneus plays an important role in the DMN (Herbet et al., 2019; Zhang et al., 2020), which is particularly important for the role of the precuneus in patients with T2DM (Yu et al., 2019), and disrupted FC in the right precuneus has been associated with insulin resistance (Musen et al., 2012). An abnormal connection between the left cerebellum and the posterior cingulate/precuneus may be a predictor of suicidal behavior in adolescents with depression (Liao et al., 2019). The association between depression and type 2 diabetes is bidirectional or comorbid, and the risk significantly increases after the age of 50 years (Roy et al., 2020). Depression scores of patients with type 2 diabetes are significantly higher than those of non-diabetic individuals, suggesting that patients with T2DM may be prone to depression. Although this study did not evaluate depression specifically, this aspect should be carefully considered in patients with T2DM who require long-term drug treatment, regular visits, strict diets, and might be more at risk for depression than other individuals.

Our analysis of correlations between FC and cognitive scores revealed that the thalamus and the right inferior parietal lobe negatively correlated with BMI, this indicates that obesity can cause serious damage to the thalamus and right inferior parietal lobe. In the NC group, fasting blood glucose levels within the normal range and higher blood glucose levels can stimulate the activity in the left precuneus brain region. The opposite of this phenomenon was observed in our diabetic patients. Thus, within normal ranges, higher fasting glucose levels have an activation effect on the left precuneus, but beyond this threshold, blood glucose levels will cause damage to the corresponding brain regions. A correlation analysis between test scales indicated that education helps with vocabulary memory and reaction ability at the beginning, but has no effect on delayed memory, suggesting that educational level does not play a significant role in cognitive assessment.

The thalamus is the higher sense center, as the most important sensory conduction replacement station of the brain. All sensory conduction pathways are projected to the cerebral cortex after neuron replacement in the thalamus. In this study, the thalamus showed a higher correlation with the test scale scores and better overall connectivity. Thalamic activation was more evident compared activation in other brain regions, and thus provides a useful metric for evaluating the progression of type 2 diabetes. Therefore, the thalamus and precuneus should be the focus of future studies on cognitive development in T2DM, given their critical role in cognitive impairment in diabetes.

### Strengths and Limitations

The present study has several strengths. First, to the best of our knowledge, this is the first study to examine the compensatory mechanism of the precuneus in conjunction with other networks in patients with T2DM before the onset of cognitive impairment, and it thus highlights the role of the precuneus network as an important neuroimaging marker in patients with T2DM. Second, by combining functional and network connectivity, we identify the regions of the brain where the differences are observed. Third, patients with cerebral infarction, including lacunar cerebral infarction, were excluded from this study to control for other interfering factors. At the same time, all main analyses were performed with age, sex, and educational level as covariates to exclude the potential influence of these factors. Compared to most earlier studies, the two groups of subjects involved in the current study were relatively young and had a narrow age range, which reduced the heterogeneity of the cohort and thereby the detrimental effect of age.

However, this study also has some limitations. First, we did not conduct a sample size estimation before the experiment. Although the sample of patients with T2DM in our study is larger than those of most T2DM studies, it is still somewhat insufficient, especially regarding the number of patients with T2DM with available cognitive domain scores. Since small sample sizes can lower the statistical power and reproducibility of a study (Chen et al., 2018), we will estimate required sample sizes and recruit more patients with T2DM in further studies that will involve multicenter patient collection. Second, we did not distinguish between patients with T2DM with subjective cognitive decline and those with mild depression. To the best of our knowledge, the severity of depression can also be associated with different levels of cognitive impairment.

## Conclusion

In summary, this study provides novel findings suggesting that higher fasting glucose level may activate the left precuneus within normal ranges in healthy individuals, which means that fasting glucose level is one of the key indicators affecting brain activation. Our findings also suggest that the precuneus network may be involved in a compensatory mechanism in T2DM-NCI, and that the thalamus, precuneus, and inferior parietal lobe may serve as sensitive neuroimaging features of cognitive impairment. Our approach provides a background for predicting cognitive deficits in patients with T2DM of different stages, and an intuitive perspective for follow-up studies on T2DM-NCI. In the future, machine learning methods could be promising for exploring the region-dependent changes in individuals with varying cognitive impairment.

## Data Availability Statement

The original contributions presented in the study are included in the article/supplementary material, further inquiries can be directed to the corresponding authors.

## Ethics Statement

The studies involving human participants were reviewed and approved by the Guangzhou University of Chinese Medicine. The patients/participants provided their written informed consent to participate in this study. Written informed consent was obtained from the individual(s) for the publication of any potentially identifiable images or data included in this article.

## Author Contributions

SK, XM, XT, HH, YF, YC, and WL contributed to the design of the study. KL, LF, JS, and JW took part in the data processing. LZ, SQ, and DH provided theoretical guidance. JW performed the data analysis and drafted the manuscript. All authors revised the manuscript and approved the submitted version.

## Conflict of Interest

The authors declare that the research was conducted in the absence of any commercial or financial relationships that could be construed as a potential conflict of interest.

## Publisher’s Note

All claims expressed in this article are solely those of the authors and do not necessarily represent those of their affiliated organizations, or those of the publisher, the editors and the reviewers. Any product that may be evaluated in this article, or claim that may be made by its manufacturer, is not guaranteed or endorsed by the publisher.

## Footnotes

1
http://www.fil.ion.ucl.ac.uk/spm

2
https://www.nitrc.org/projects/gretna/

3
http://icatb.sourceforge.net
